# Epigenomic mediation after adverse childhood experiences: a systematic review and meta-analysis

**DOI:** 10.1080/20961790.2019.1641954

**Published:** 2019-08-26

**Authors:** Inês Neves, Ricardo Jorge Dinis-Oliveira, Teresa Magalhães

**Affiliations:** a Department of Public Health and Forensic Sciences, and Medical Education, Faculty of Medicine, University of Porto, Porto, Portugal; b IINFACTS-Institute of Research and Advanced Training in Health Sciences and Technologies, Department of Sciences, University Institute of Health Sciences (IUCS), CESPU, CRL, Gandra, Portugal; c UCIBIO, REQUIMTE, Laboratory of Toxicology, Department of Biological Sciences, Faculty of Pharmacy, University of Porto, Porto, Portugal

**Keywords:** Forensic sciences, forensic genetics, child abuse, DNA methylation, epigenetics, meta-analysis, mental illness, HPA axis

## Abstract

Epigenetic mechanisms are potential mediators of the physiological response to abuse by altering the genetic predisposition of the cellular response to the environment, leading to changes in the regulation of multiple organ systems. This study was established to review the epigenetic mechanisms associated with childhood abuse as well as the long-term determinants that these epigenetic changes may have on future illness. We retrospectively analysed the effect of exposure to adverse childhood experiences (ACEs, specifically those relating to childhood maltreatment) between the ages of 0 and 16 years on the human epigenome, as well as possible clinical associations. After meeting inclusion and exclusion criteria, 36 articles were included in this systematic review. Eight of these studies did not find a relationship between childhood maltreatment and DNA methylation. Of the remaining 28 studies, nine were genome-wide association studies, whereas the rest were candidate gene studies, mainly studying effects on neuroendocrine, serotoninergic and immunoregulatory systems. Meta-analysis of correlation coefficients from candidate gene studies estimated an association of childhood adversity and DNA methylation variation at *r* = 0.291 (*P* < 0.0001), and meta-analysis of two epigenome-wide association studies (EWASs) identified 44 differentially methylated CpG sites. In conclusion, childhood maltreatment may mediate epigenetic mechanisms through DNA methylation, thereby affecting physiological responses and conferring a predisposition to an increased risk for psychopathology and forensic repercussions. Similar evidence for somatic illnesses is not yet available.

**KEY POINTS**
- Adverse childhood experiences are associated with increased mortality partly explained by acquired epigenetic changes
- There is a positive correlation between childhood abuse and DNA methylation at specific gene sites
- The cumulative effect of different types of childhood abuse and neglect may lead to changes in DNA methylation
- Epigenome changes associated with childhood abuse appear to be involved in the development of psychiatric illness in adulthood
- Studying epigenetic changes may have important public health and forensic applications in the future

## Introduction

The adverse childhood experiences (ACEs) study is a seminal work that laid the foundation for research into the long-term effects of child abuse and household dysfunction, in particular by increasing the risk of mortality due to ischaemic heart disease, cancer, chronic lung disease, liver disease and fractures [1]. A recent meta-analysis showed that individuals with at least four ACEs had an increased risk of all negative health outcomes, with strong associations for sexual risk-taking, mental ill health, problematic ethanol abuse, problematic drug use and violence, both interpersonal and self-directed [2]. Numerous studies have revealed associations between childhood maltreatment and psychopathology [3], cardiovascular illnesses [4], autoimmune disorders [5] and cancer [6].

A commonly accepted explanation for the association between childhood adversity and increased disease risk relies on the use of coping behaviours such as smoking, illicit drug use and risky sexual behaviours [1], which are well-established risk factors for disease. Another explanation examines the biological effects of the traumatic stress response, which involves the chronic activation of the sympathetic nervous system and the hypothalamic–pituitary–adrenal (HPA) axis, leading to neuroendocrine and immune disruption [7, 8] and altered systemic cortisol production [9]. Structural alterations of the central nervous system, namely, in the inferior frontal orbital gyrus [10] and decreased hippocampal size in suicide cases [11], have been described in patients exposed to childhood maltreatment [12]. Moreover, the importance of infantile experience and early exposure to stress has long been established in animals [13] and evidence is rapidly growing for the role of epigenetic modifications in both animal and human models of trauma and abuse [14].

Epigenetic mechanisms are potential mediators of the physiological response to abuse by altering the genetic predisposition of the cellular response to the environment, eventually leading to changes in the regulation of multiple organ systems at the most basic level [15, 16]. DNA methylation creates cell-type identity by adding methyl groups to cytosines at distinct points in the genome through stable covalent bonds [17] and silences the expression of these genes when methylation occurs at promoter sites [18]. Increasing evidence has identified the importance of DNA methylation of genes related to glucocorticoid regulation in the aetiology of various diseases and underlined the importance of the HPA axis in regulating social and environmental stressors [19].

In the literature, there are attempts to summarize the impact of ACEs, socioeconomic position and early environmental stressors on the human genome and future risk of disease [20–22], in some cases describing the transmission of trauma across generations [23]. Few studies, however, have considered the epigenetic modification of genes outside of the HPA axis or even the cumulative impact of abuse. In several studies, measurements of types of psychosocial adversity that may or may not be considered abusive (such as early institutionalization, adoption or low socioeconomic status [24, 25]) were performed; earlier work could also have been limited by the use of invalidated tools to measure exposure to ACEs. In terms of epigenetic changes related to childhood adversity, studies have often disagreed on the direction and magnitude of the effects, even for well-studied genes [12]. At present, there is no comprehensive review summarizing the epigenetic mediation of childhood maltreatment across the genome while comparing the statistical evidence and the clinical outcomes associated with these effects.

This study thus aims to summarize the epigenetic mechanisms associated with childhood abuse as well as the long-term effects that these epigenetic changes may have on future illness. More specifically, this study was established to perform a meta-analysis of epigenome-wide association studies (EWASs) to quantify the effect that these ACEs may have on the epigenome, as well as a separate meta-analysis to study whether differentially methylated genes could be used as potential biomarkers for childhood maltreatment, with clinical and forensic implications.

## Methods

The systematic review and meta-analysis were carried out in accordance with the Cochrane Preferred Reporting Items for Systematic Reviews and Meta-Analyses (PRISMA) guidelines [26]. Five databases were used to retrieve data for this review without a limiting period: PubMed (311 results), EBSCOHost (269 results), ISI Web of Science (56 results), Scopus (284 results) and ScienceDirect (122 results). Articles presenting an original study design were included, and reference lists of relevant reviews were searched. The search query used for PubMed may be found in Appendix I. In this search query, terms relating to childhood trauma, childhood adversity and familial adversity were used to determine exposure to ACEs. Terms including the acronym ACE but not focussing on child abuse and victimology were excluded from the search. Articles analysing the effects of prenatal or familial stress, low socioeconomic status and institutionalization alone were excluded since those studies exclusively focused on genetic polymorphisms. It was assumed that DNA methylation levels would be equivalent irrespective of the tissue sample used. Therefore, studies were included if they: i) were performed on adult human subjects (over the age of 16), ii) used a validated method for ascertaining exposure to childhood trauma, and iii) included a relevant control group (healthy and/or at low risk for childhood abuse).

Articles were screened at the title/abstract level to filter out unoriginal study designs and irrelevant articles. Full-text screening was performed for the remaining 90 articles, with 36 articles being included after discussion among the authors. The Newcastle–Ottawa Scale for assessing the quality of nonrandomized studies [27] was applied. Studies fulfilling the inclusion criteria and presenting a method for analysing DNA methylation across the genome using the Illumina HumanMethylation450 BeadChip were considered for inclusion in an EWAS meta-analysis. Authors were contacted and asked to provide summary data or the raw methylation data files for their EWASs, if these were not publicly available as Gene Expression Omnibus (GEO) datasets. Meta-analysis was performed in METAL using an *P*-sample size-based fixed-effects model with genomic control correction [28]. Probes surviving a false discovery rate (FDR) correction of 5% were considered significant, with a *P*-value threshold set at *P* < 0.0001 for this study. In addition, pathway analysis of significant CpGs was undertaken using ConsensusPathDB [29].

To study whether candidate genes could be used as biomarkers for child abuse, the *metafor* package in R was used. The effect size used was Pearson’s *r* correlation coefficient measuring the association between DNA methylation of certain loci and childhood trauma, with the modulus of *r* (|*r*|) used to represent the strength of differential methylation variation. A random-effects model was applied for all analyses. When no data were reported for a nonsignificant association, the authors assigned *z* = 0.00 as a conservative estimate.

## Results

**Figure 1.**
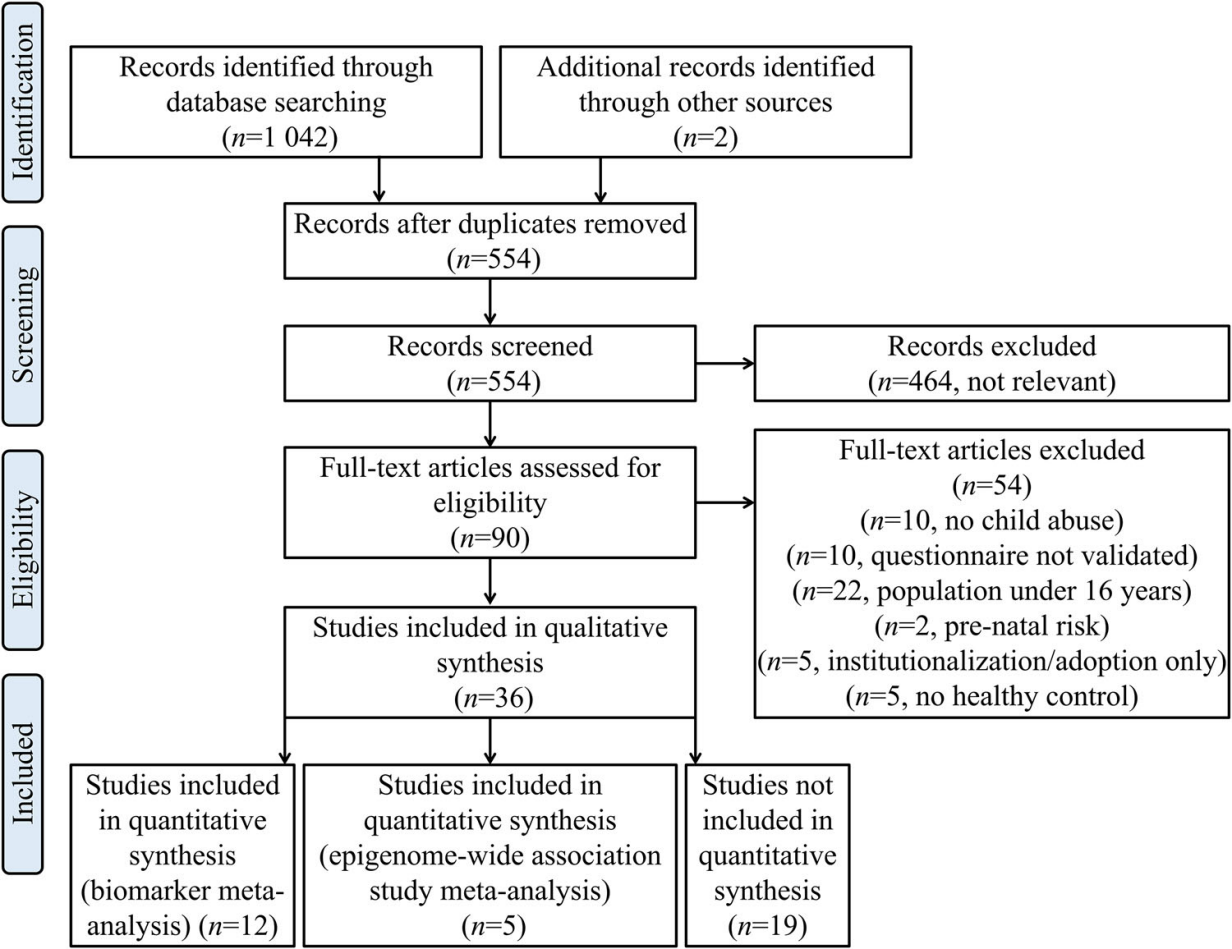
Flow diagram of study selection according to Preferred Reporting Items for Systematic Reviews and Meta-Analyses (PRISMA) guidelines.

A flow chart of the selection process is presented in Figure 1. Briefly, after meeting the inclusion and exclusion criteria, 36 articles were included in this systematic review. Of these, five articles were included in a meta-analysis of EWASs, and 12 studies were further included in a child abuse biomarker meta-analysis. A summary of the DNA methylation changes along with an evaluation of study quality is shown in Table 1. Studies using searches for “neoplasms”, “autoimmune diseases” or “cardiovascular diseases” were not registered.

**Table 1.** Summary of DNA methylation changes associated with childhood adversity.

Gene	Direction of change	Notes	Quality score^a^	References
*NR3C1*	Hyper-methylation	Differential methylation in central nervous tissues, but inconclusive in peripheral tissues	5.4/10	[15, 40, 42, 45, 46]
			6/13	[41]
*FKBP5*	Hypo-methylation (rs1360780 allele)	Increased severity of depressive symptoms; greater risk for adulthood PTSD	5/10	[42, 48]
			6/13	[10, 47]
*SLC6A4*	Hyper-methylation	Associated with major depressive disorder and childhood sexual and physical abuse	5/10	[50, 51]
			8/13	[49]
*BDNF*	Hyper-methylation	Global methylation related to total childhood trauma and physical abuse in samples with comorbid borderline personality disorder	5/10	[52, 53]
*DRD2*	Hyper-methylation	Childhood sexual abuse compounded with bulimia spectrum disorder	4/10	[54]
*IL6*	Hypo-methylation	Greater *IL6* concentrations and reduced cortisol response	NA	[55]
*MAOA*	Hyper-methylation	Sexual abuse and depression was associated with hyper-methylation of *MAOA* first exon	5/13	[56]
*OPRK1*	Hypo-methylation	Decreased hydroxymethylation of intron 2 in the Kappa variant 1 region	5/10	[57]
*OXTR*	No change	Site-specific CpG methylation and child abuse predicted adulthood depression and anxiety	NA	[58]
*SKA2*	No change	*SKA2* methylation levels and exposure to childhood abuse predicts risk of future PTSD	5/13	[59]
*SSTR4*	No change	Increased in subjects with ethanol dependence	6/10	[60]
*MT-ND6*	Hyper-methylation	Greater methylation and blunted cortisol response in subjects with ≥4 ACE categories	NA	[61]
*rRNA*	Hyper-methylation	Restricted to the hippocampus of suicide victims with a history of child abuse	6/10	[11]
*LINE-1* sequence	Hypo-methylation	Lower methylation predicted by higher emotional abuse and general trauma scores	4/10	[62]
*BAGE* sequence	No change	Higher total trauma scores predicted *BAGE* methylation	4/10	[62]

^a^ Quality of evidence was determined using the Newcastle–Ottawa Scale (rating refers to number of stars out of 10 for case-control studies and out of 13 for cohort studies). PTSD: post-traumatic stress disorder; NA: scale not applicable; ACE: adverse childhood experience.

### Genome-wide association studies

There are nine genome-wide studies among the 36 articles included. DNA methylation of the central nervous system at the genome-wide level was only examined in one study. Labonté et al. [30] used hippocampal samples from 25 suicide victims with a history of child abuse and 16 suicide victims with no history of child abuse and found 362 promoters differentially methylated in the former group (68.5% of which were hyper-methylated), with changes being equally distributed throughout the genome. O’Donnell et al. [31] used data from a prospective cohort and randomized controlled trial of a psychosocial intervention programme (Nurse Family Partnership) to measure global variation in DNA methylation 25 years after the intervention had come to an end. In that study, child abuse and neglect were associated with approximately 13.6% of genome-wide variation in methylation between individuals in adulthood.

In another study [32], gene expression profiles of post-traumatic stress disorder (PTSD) patients were found to be almost completely distinct: differential methylation of CpG sites was shown in 69.3% of transcripts in cases with child abuse, but only 33.6% of transcripts in cases without child abuse were differentially methylated. Lawn et al. [33] found that subjects with a history of childhood sexual abuse had a higher DNA methylation age (an increase of 3.41 years at the 47-year time point). In addition, a cross-sectional study by Zhang et al. [34] studied 82 candidate genes in 518 individuals and found that exposure to childhood abuse in European-Americans was associated with differential methylation in both subjects with ethanol dependence and controls, while no site was differentially methylated in African-Americans. In a group of former child labourers, 71 differentially methylated CpGs were found [35].

Cecil et al. [36] studied a group of 124 ethnically diverse individuals from low-income areas of London, with 68% of individuals reporting at least one form of maltreatment. Results also evidenced the greatest number of differentially methylated probes for physical neglect (118 probes), followed by physical abuse (34 probes) and sexual abuse (7 probes).

Houtepen et al. [37] performed an EWAS regarding the relationship between cortisol reactivity and DNA methylation in a sample of 85 individuals. They found a significant association between childhood maltreatment and decreased cortisol stress reactivity in a locus in *KITLG*, which mediated the relationship in 32% of Caucasians.

Finally, only one genome-wide association study of the nine studies included in this review found no changes relating DNA methylation and child abuse, regardless of whether a genome-wide or site-specific level was considered [38].

### Meta-analysis of genome-wide association studies

**Figure 2.**
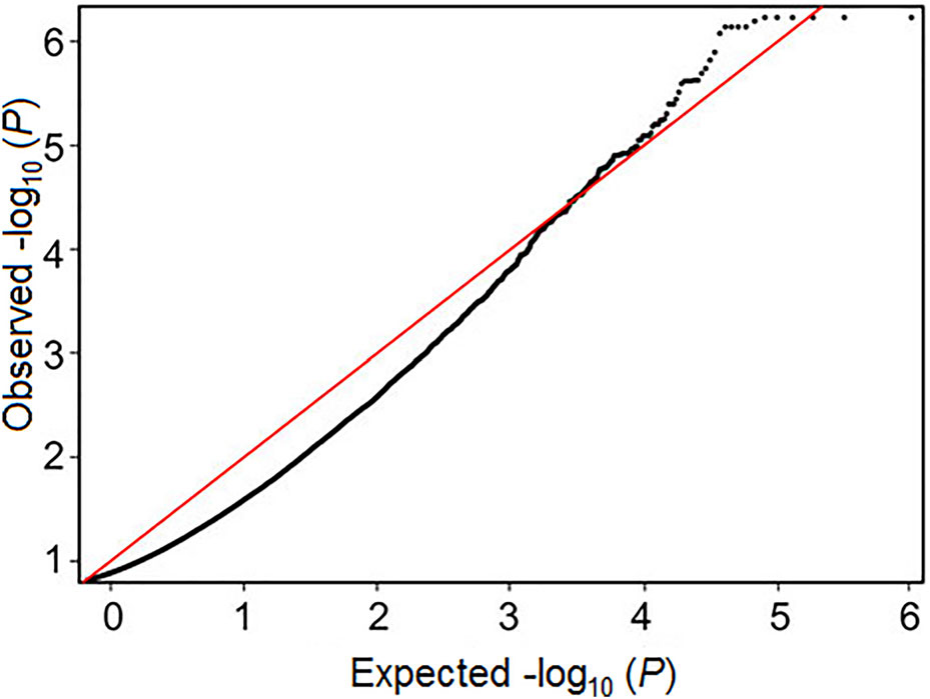
QQ-plot for epigenome-wide association study (EWAS) meta-analysis.

**Figure 3.**
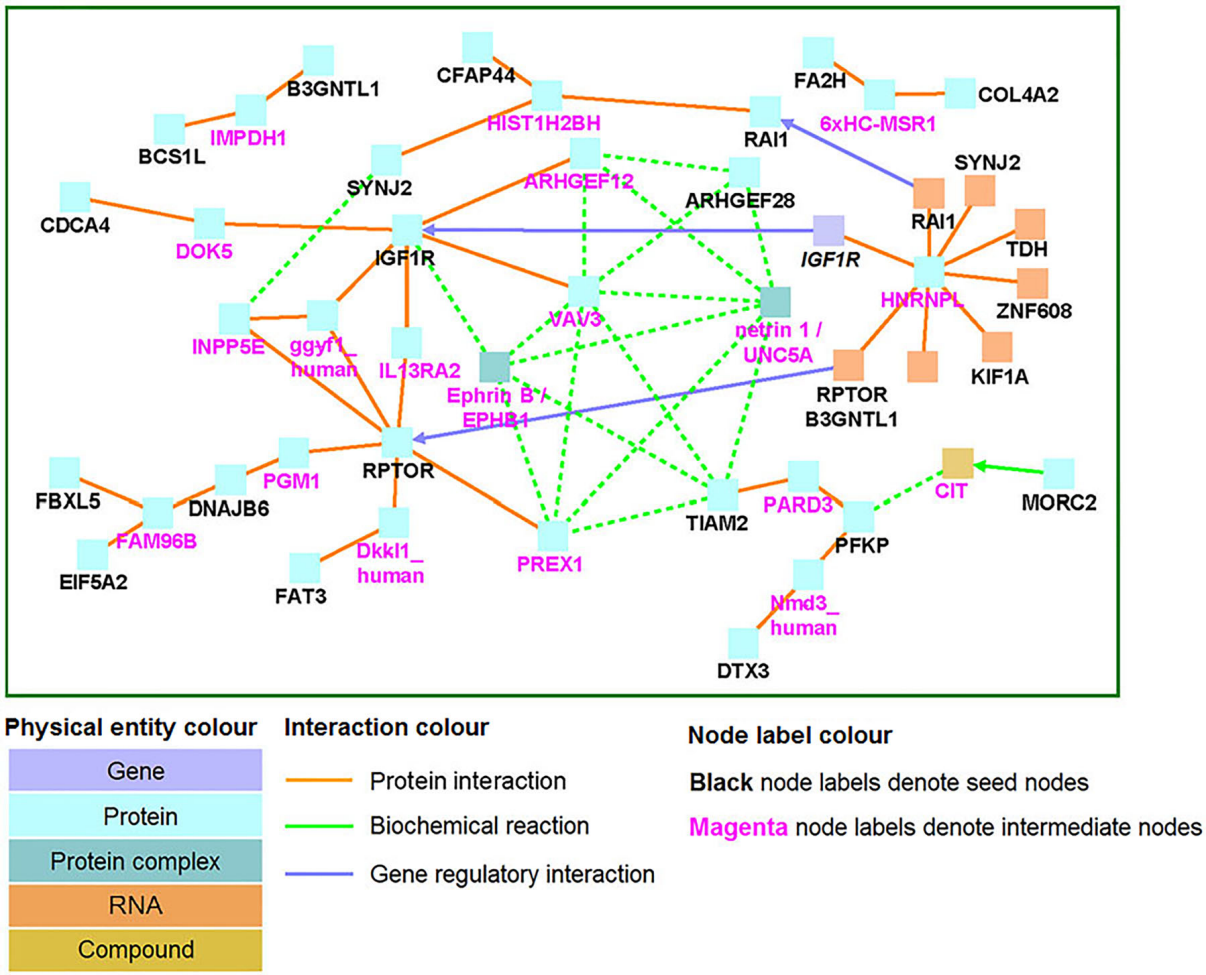
Induced Network Module Analysis for genes mapped to differentially methylated CpG sites (ConsensusPathDB).

Of the nine genome-wide association studies, five articles utilized a common method (Illumina HumanMethylation450 BeadChip) to analyse DNA methylation across the genome, which made them eligible for inclusion in a meta-analysis of EWASs [31, 32, 35–37]. Cecil et al. [36] kindly made summary data from their study available, and discovery data from Houtepen et al. [37] were accessed from the GEO data repository (GEO Accession Number: GSE77445). Data were run in METAL using a *P*-sample size-based approach, leading to 44 CpGs being considered significant for this meta-analysis, 97.7% of which were hypo-methylated. The full sample size included 209 individuals, with moderate heterogeneity (mean I^2^ = 58%). A QQ-plot for the FDR-corrected values showed an approximately normal distribution (Figure 2). Two CpG sites were situated near the *RPTOR* gene, which regulates cell growth in response to nutrient and insulin levels. The results of network analysis are shown in Figure 3. The genes identified are associated with molecules in the PI3K-AKT signalling pathway (RTK, ECM, ITGB and Raptor) and in the AMPK signalling pathway (PFK-1, IGF1R and mTORC1). Gene ontology analysis found the enrichment of biological processes primarily associated with nervous system development and the regulation of multicellular organismal processes.

#### NR3C1

Nine studies explored the consequences of childhood maltreatment on the glucocorticoid receptor. Labonté et al. [39] examined the hippocampus and anterior cingulate cortex in a sample of suicide completers with a history of child abuse, suicide completers with no history of child abuse and control subjects who died of natural causes. This study described differential, site-specific methylation of the hGR1B and hGR1C promoter, while the hGR1H promoter was the only region with reduced mean methylation in the hippocampus of suicide victims. Another study also used hippocampal samples and found that suicide victims with a history of child abuse had decreased levels of glucocorticoid receptor mRNA as well as increased methylation at specific sites of exon 1 F [40]. Other studies relied on peripheral samples to study epigenetic changes. One cohort study using 152 individuals from the Detroit Neighborhood Health Study associated childhood maltreatment with increased DNA methylation in an EGR1 transcription factor binding site, as well as increased methylation of CpG sites 1–4 of *NR3C1* [41]. In a case-control study, Farrell et al. [42] found a significant association between the severity of emotional abuse in childhood and increased methylation at *NR3C1* exon 1 F in a subgroup of 33 depressed patients who reported early life adversity.

Two cross-sectional studies designed by the same group of researchers found contrasting results. In the first study, Tyrka et al. [43] recruited a sample of 99 healthy participants and found increased *NR3C1* promoter methylation at CpG1 and CpG3 in response to adversity. In contrast, childhood adversity was associated with decreased *NR3C1* promoter methylation in a separate group of 340 adults [44]. Three other studies found no association between childhood abuse and DNA methylation of *NR3C1* [15, 45, 46].

#### FKBP5

Four studies focused on the *FKBP5* gene and its relationship with PTSD and major depressive disorder (MDD). Two studies did not find any relationship between *FKBP5* methylation and adversity or between depressed patients and controls [42, 47]. In contrast, a cohort analysis in Ireland found that childhood adversity predicted hypo-methylation of *FKBP5* in patients with MDD carrying a high-risk allele (rs1360780), which also correlated with reduced grey matter concentration in the inferior frontal orbital gyrus. Decreased activation in this region was also found to be related to the severity of depression [10]. Another study examined the association between childhood abuse and PTSD, describing a strong association between individuals carrying the same risk allele (rs1360780) and increased risk for adulthood PTSD after childhood, but not adulthood, trauma. Those exposed to childhood trauma and carrying the risk allele had, on average, a 12.3% decrease in methylation of a specific *FKBP5* region [48].

#### SLC6A4

Three studies examined the effect of childhood maltreatment on methylation of the *SLC6A4* gene. In a group of 26 women reporting a history of child sexual abuse, symptoms of antisocial personality disorder, depression and substance abuse were more prevalent and methylation of the CpG island surrounding exon 1 of *SLC6A4* was increased [49]. Two case-control studies also studied the relationship between MDD and childhood trauma. Okada et al. [50] found separate differentially methylated sites associated with the total number of adversities (specifically, hyper-methylation of CpG76 and hypo-methylation of CpG3). In a smaller study of 69 Irish individuals, 30 of whom had a diagnosis of MDD, childhood trauma (specifically physical abuse), but not depression, was significantly associated with *SLC6A4* methylation [51].

#### BDNF

Two studies examined the effect of childhood maltreatment on the *BDNF* gene. Perroud et al. [52] found higher Childhood Trauma Questionnaire (CTQ) scores in borderline personality disorder (BPD) outpatients and a greater total percentage of *BDNF* methylation was associated with increased childhood trauma. A separate analysis found that women with bulimia nervosa who suffered physical abuse in childhood had a greater percentage of DNA methylation across all CpGs than women without eating disorders, whereas childhood sexual abuse in bulimic patients was associated with differential methylation of specific *BDNF* sites [53].

### Other candidate genes

Several studies looked at other candidate genes to describe any epigenetic changes associated with childhood maltreatment, specifically in regions governing pain sensitivity, immune response and neurotransmission. For example, the *DRD2* gene was studied in a group of Canadian women, most of who suffered from bulimia spectrum disorder, a condition that when compounded with childhood sexual abuse was associated with higher methylation levels [54]. In addition, a cross-sectional study in 34 African-American men found that exposure to childhood trauma as measured by the CTQ led to significant hypo-methylation of the *IL6* promoter. The group exhibiting *IL6* hypo-methylation had a reduced cortisol response and prolonged *IL6* elevation after stressful stimuli [55]. Moreover, Checknita et al. [56] designed a prospective study with a 75-month follow-up of 114 Swedish women. Those who had suffered childhood physical and/or sexual abuse had higher methylation of the *MAOA* gene. Physical abuse was particularly associated with hyper-methylation at CpG7/8, whereas sexual abuse was particularly associated with hyper-methylation of the *MAOA* first exon as well as an increased risk of current depression.

Lutz et al. [57] studied methylation of the kappa opioid receptor (*OPRK1*) using brain tissue of suicide victims with and without a history of child sexual and physical abuse and neglect compared with a group of psychiatrically healthy victims who had died of accidental causes. Methylation of intron 2 in the Kappa variant 1 region was significantly lower in child abuse suicide cases, which was related to decreased kappa expression in the anterior insula.

In addition, *OXTR* methylation was studied in a group of 393 African-American subjects. This study described an interaction between child abuse and CpG methylation, which was predictive of both depression and anxiety [58].

Boks et al. [59] did not find any relationship between childhood trauma and *SKA2* methylation in a group of 85 healthy controls. However, pre-deployment *SKA2* methylation levels and childhood trauma exposure did significantly predict post-deployment PTSD symptoms in a cohort of 94 military servicemen.

Moreover, a case–control study on 176 Polish individuals with ethanol dependence and 127 healthy volunteers found that the former group reported significantly higher numbers of childhood adversities. However, childhood trauma was not found to be a predictor of *SSTR4* methylation, despite 21.6% of this population displaying increased *SSTR4* promoter methylation compared with 2.3% of controls [60].

A further study considered epigenetic changes in mitochondrial DNA, finding significantly higher average *MT-DN6* (NADH dehydrogenase 6) methylation levels in a group of 42 individuals with an ACE score ≥4, an epigenetic change also associated with higher baseline cortisol levels and blunted cortisol response to stress [61].

Repeating DNA sequences *LINE-1* and BAGE were also studied in 48 patients presenting with first-episode schizophrenia and 48 healthy controls. Schizophrenic patients with a history of child abuse had significantly lower *LINE-1* methylation, which was predicted by higher emotional abuse and total trauma scores [62].

Finally, in an earlier study by McGowan et al. [11], *rRNA* promoter methylation was studied in cerebral samples from 13 individuals with a history of child abuse who had committed suicide and 11 sudden death controls. The former group had greater *rRNA* promoter methylation in hippocampal tissue.

### Meta-analysis of candidate gene studies

**Figure 4.**
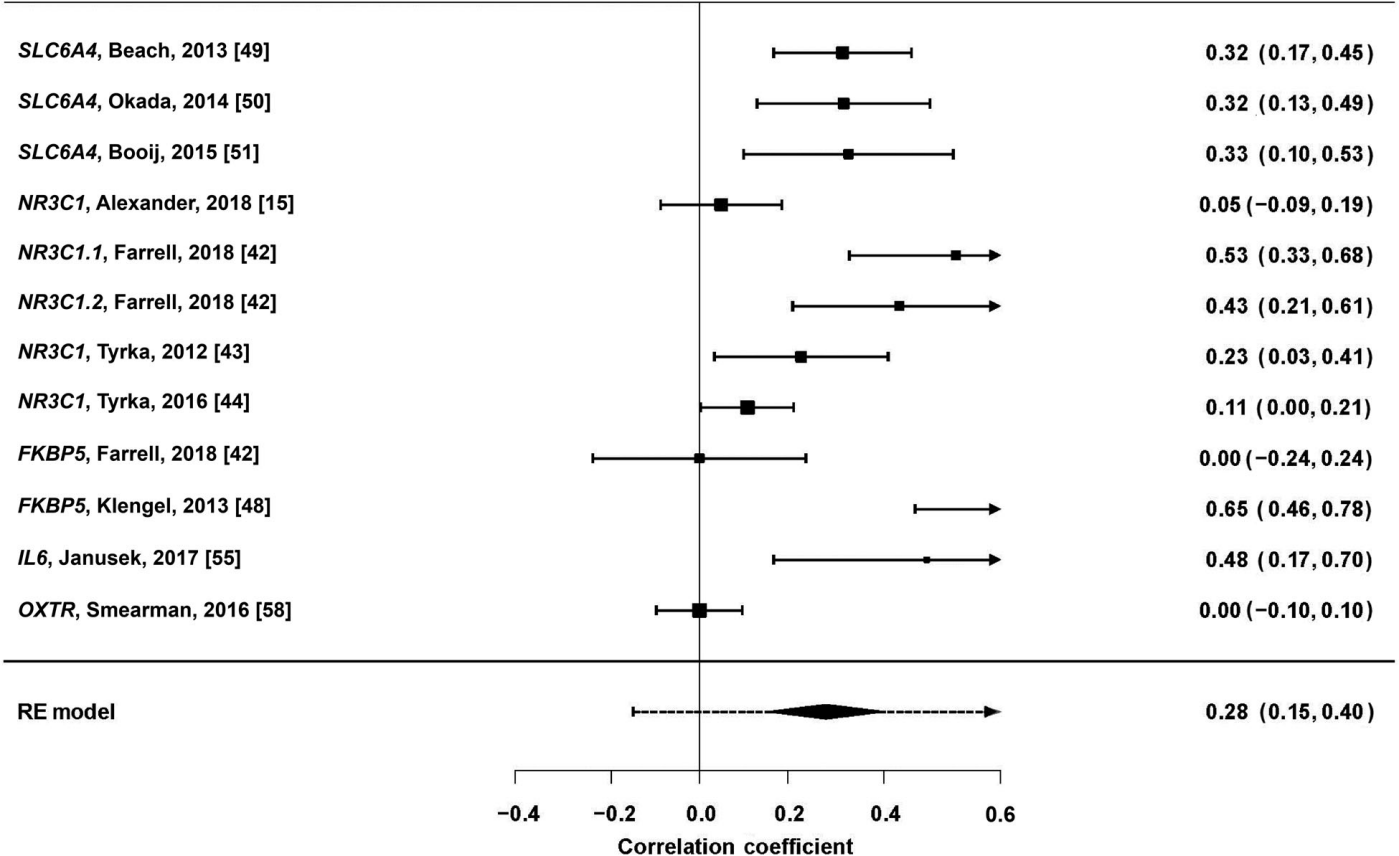
Forest plot of candidate gene methylation variation (random-effects (RE) model using |*r*| correlation coefficients).

**Figure 5.**
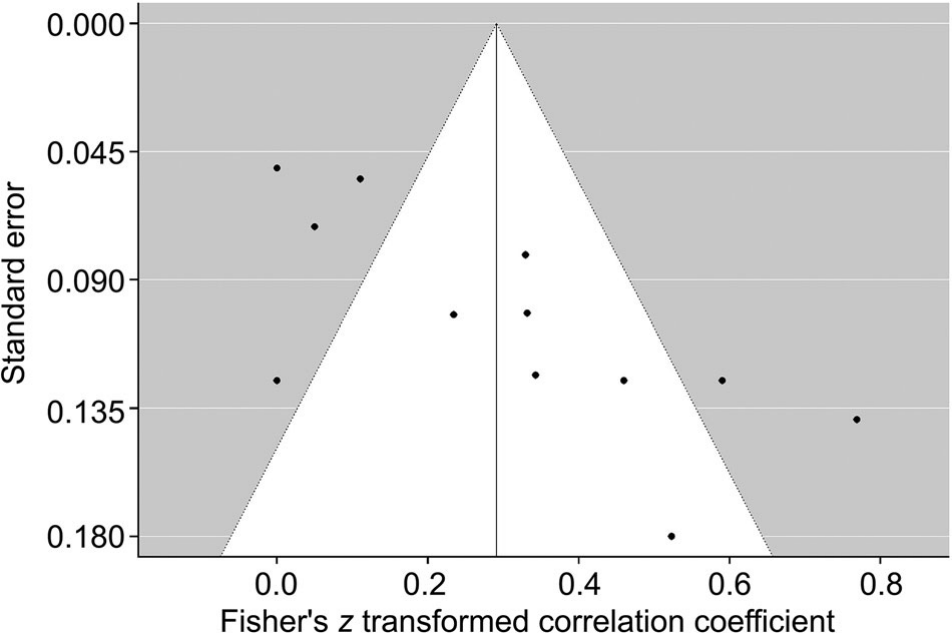
Funnel plot of studies included in candidate gene meta-analysis.

A random-effects meta-analysis of studies reporting *r* correlation coefficients was undertaken for *SLC6A4*, *NR3C1*, *FKBP5*, *IL6* and *OXTR*. Correlation coefficients for four studies [15, 44, 48, 55] were changed from negative to positive associations, as the aim of this meta-analysis was to determine the strength of differential methylation. Studies reporting a nonsignificant effect had their correlations estimated at *z* = 0.00 [42, 58]. Risk allele correlations were used for the study by Klengel et al. [48]. The overall weighted correlation coefficient across all 12 measurements was *r* = 0.291 (*P* < 0.0001), although examination of the forest plot (Figure 4) suggested a trend-level association. A random-effects model was deemed appropriate as heterogeneity between studies was elevated (I^2^ = 85.23%). Egger’s regression test indicated the presence of publication bias (*z* = 3.5475, *P* = 0.0004) (Figure 5). Subgroup analysis for *SLC6A4* found a larger effect for DNA methylation variation (*r* = 0.3329, *P* < 0.001), while analysis for *NR3C1* gave a less significant result (*r* = 0.2692, *P* = 0.0075).

## Discussion and future perspectives

This review strongly suggests that epigenetic changes throughout the genome have a positive, long-lasting relationship with childhood abuse and adversity, with possible clinical and forensic repercussions. These changes tend to be both gene-specific and specific to the type of childhood trauma experienced, with the strongest association for total trauma scores, followed by sexual and physical abuse in childhood [3]. It is also clear that EWASs are increasingly common and fundamentally useful to evaluate the epigenetic variation at the genome-wide level and associate these changes with specific phenotypes. In the studies under review, epigenetic variation was generally studied to measure changes in the cortisol response to stress tasks such as the TSSR, and often identified a history of childhood trauma as a factor modifying cortisol levels. Except for one study [38], childhood adversity was found to have effects on the methylation status of various loci across the genome.

Induced network module analysis (Figure 3) of the genes containing differentially methylated loci in the meta-analysis of two EWASs identified numerous biochemical and protein interactions between the genes expressed (except for B3GNTL1 and BCS1L, which encode an acetylglucosaminotransferase enzyme and a mitochondrial enzyme, respectively [63]). These lines of evidence that childhood maltreatment may influence numerous interrelated pathways and the identification of gene ontology associations between differentially methylated genes and neuronal growth pathways and regulation of multicellular processes point to widespread organismal effects.

The *NR3C1* gene encodes the glucocorticoid receptor involved in inflammatory responses and cellular proliferation and differentiation in targeted tissues through the HPA axis, a major neuroendocrine system responsible for regulating the stress response through numerous biological feedback mechanisms [63]. Interestingly, this gene presents a unique structure with eight coding exons and a noncoding region consisting of nine alternative first exons, each of which has its own promoter [19]. This region is responsible for transcriptional regulation of the glucocorticoid receptor, which may explain the variation in results reported in this review, such that only studies in brain tissues reported a common effect (hyper-methylation of the *NR3C1* gene at specific loci). *FKBP5* was only found to be hyper-methylated in individuals with a high-risk T allele, underlining the importance of genotyping when certain phenotypes have known genetic risk profiles. This gene encodes an immunophilin protein, which participates in immunoregulation as well as protein folding and trafficking within the cell [63]. *SLC6A4* encodes a membrane protein responsible for the transport of serotonin across the synaptic space [63]. A systematic relationship between DNA methylation and childhood abuse was found for *SLC6A4*, providing a basis for a gene environment interaction between trauma and depression [51]. *BDNF*, a gene encoding a protein involved in nerve growth [63], has been implicated in the aetiology of BPD, and as such was studied in populations suffering from this disorder, which found that this gene was globally methylated in patients with a history of childhood trauma [52].

Although less evident, other candidate genes are also promising regions for further study as these genes are often related to disease phenotypes. Indeed, *DRD2*, *IL6*, *MAOA* and Kappa genes showed differential methylation profiles, some of which were associated with bulimia nervosa and depression [54, 56, 57].

It is also becoming evident that a number of research groups are interested in using these differentially methylated genes as clinically meaningful prognostic biomarkers [19], such as predictors of suicide in sufferers of PTSD [59]. Indeed, this review analysed several studies showing strong associations between childhood abuse and future risk of depression, anxiety and PTSD [10, 32], as well as intensified symptoms of borderline personality disorder, bulimia nervosa and ethanol dependence [45, 54, 60]. Identifying these types of trauma may be useful to stratify patient populations at increased risk of developing symptomatology and poor treatment response [50, 52]. Childhood abuse, even if not directly responsible for the epigenetic profiles associated with these pathologies, may mediate or even predict their appearance [59] and as such the presence or absence of risk factors for ACEs should be documented in clinical practice. Moreover, knowing that victims of childhood abuse are generally associated with poorer treatment outcomes for psychiatric disease [64], subgrouping patients based on favourable genetic biomarkers may lead to more effective treatment options. Considering that DNA methylation may be reversible [13], studying these epigenetic marks may lead to novel therapeutic approaches, whether through pharmacological [65] or psychosocial interventions [31]. Nevertheless, these results should be interpreted with caution, as publication bias and the small-study effect may have inflated this correlation, and differential methylation in many cases refers to specific CpGs or types of trauma. Moreover, larger-scale studies are needed to better examine this association and the included studies exhibit a high level of heterogeneity among them (I^2^ = 85%), which was accounted for in a random-effects model, recognizing the increased probability of sampling and effect variability in the populations studied.

The evidence base of the studies included in this analysis is moderate in quality, considering the parameters outlined in the Newcastle-Ottawa Scale (Table 1). This may be explained by the cross-sectional measurements of epigenetic alterations, despite many studies using cohort or case–control designs. Common identified limitations include: recall bias due to retrospective application of questionnaires such as the CTQ; small sample sizes leading to a lack of statistical power; lack of data on factors known to alter DNA methylation (smoking and medication use); use of peripheral tissues to measure changes in neural processes; exposure to multiple types of trauma with difficulty in determining whether changes were due to a specific ACE; and the presence of possible confounding factors (such as nutrition and varying cell types in whole blood and saliva). As such, the results presented here should be interpreted with caution and these limitations should be addressed in future research. Considering that reported studies used different cohorts for their samples, there may be interest in using a series of measurements to determine whether the epigenetic profiles of trauma victims change over time, how these changes may relate to a patient’s disease course and to determine a causal role for certain epigenetic changes. Moreover, it should be highlighted that, regarding the sociodemographic characterization of the studied populations, 13 studies did not specifically report information on ethnicity. Although several studies were based on minority or disadvantaged populations, care should be taken to characterize the environmental and socioeconomic stresses present in these populations.

A major difficulty in interpreting candidate gene studies is the lack of consistency in nomenclature of the genetic regions under study, especially when discussing differentially methylated loci. Comparing findings is also difficult when the studied regions vary significantly among studies focussing on the same gene, with some studies looking at the whole promoter region, while others focus on specific CpG islands. The genetic regions chosen should be selected based on previously studied regions, while researchers who wish to study novel sites should include loci overlapping with those from previous research to aid comparison. It should be noted that disease associations may be specific to a certain genotype, as seen for the *FKBP5* high-risk T allele rs1360780 [10, 48]. Research should also consider risk genotypes when investigating outcomes or exposures with known genotype associations.

Although increased DNA methylation of a gene is generally associated with decreased expression, it is of fundamental importance that future studies both measure gene expression and quantify associated levels of hormones or biomarkers. These functional results are essential to understand whether certain loci are sensitive to a determined exposure or pathology and may help to explain results that appear contradictory. Research should also include CpG sites located in transcription factor binding sites, as it is through inhibition of these sites that DNA methylation silences genetic expression [19].

The tissues used in these studies may be responsible for the variation in the obtained results regarding epigenetic changes. As changes after childhood trauma are generally associated with neural pathways, studies utilizing brain or neuronal tissue should be favoured over those using peripheral measures. The use of peripheral blood may bring further challenges, aside from possibly not being representative of central tissues (although correspondence has been documented in gene expression signals in both brain and blood tissues [66]). A possible confounding factor is the heterogeneous cell populations in whole blood, which can be resolved by statistical adjustment for the particular cell composition in blood samples. However, only six of the studies in this review included statistical adjustments for cell count.

A clear limitation of this meta-analysis is that the authors are not part of a consortium, as is usual for meta-analyses of genome-wide association studies, allowing the results to be pooled across multiple cohorts. Although an attempt was made to contact the authors of five articles considered for an EWAS meta-analysis, only two authors made their data available for use in this study. Another limitation is the existence of publication bias in the candidate gene meta-analysis, most likely due to the small-study effect.

Despite these limitations, different ACEs appear to have different effects on the epigenome, especially in the *SLC6A4* gene. Future studies should consider the differing effects that certain types of adversity may have on the epigenome, as well as on the presentation of disease, and should aim to characterize each of these ACEs separately. Another major gap in research is apparent. Despite ACEs being associated with changes in several genes with effects on various physiological processes, studies identified using the search query outlined in this review only examined psychiatric illness. Even using search parameters specifying somatic diseases such as “neoplasms”, “autoimmune diseases” or “cardiovascular diseases” (illnesses strongly associated with childhood trauma), no study definitively relating epigenetic changes after childhood adversity with any of these pathologies in humans was identified, even though correlations with somatic illnesses may be inferred through known epigenetic modifications of the HPA axis and immune system.

Acknowledging the importance of prevention in reducing the nefarious effects of childhood abuse at both the individual and societal levels, studying these epigenetic effects and clinical outcomes may lead to more effective public health policies, interventions and better forensic decisions based on trauma-informed care models [8, 19]. On a more practical level, studying the effects of abuse on the epigenetic clock may lead to more accurate methods of dating individuals, as seen in the work by Lawn et al. [33], in which childhood sexual abuse led to an average increase in DNA methylation age of 3 years. More importantly, these studies form a basis for epigenetic mediation of numerous physiological processes in the stress response, as well as a biological association between childhood trauma and the increased risk for several types of psychopathology, especially depression and PTSD. However, several important limitations must be addressed in future epigenetic studies in order to produce a more robust evidence base for future public health policies and trauma-informed interventions in healthcare to reduce the nefarious long-term effects of childhood trauma at both individual and societal levels.
